# Metformin improves pregnancy outcomes in non-PCOS women with insulin resistance and recurrent implantation failure before frozen embryo transfer

**DOI:** 10.3389/fendo.2025.1671899

**Published:** 2025-12-10

**Authors:** Liying Peng, Wanli Yang, Mengyang Du, Xujing Deng, Ruixiu Zhang, Dengke Qin, Shihua Bao

**Affiliations:** 1 Department of Reproductive Immunology, Shanghai First Maternity and Infant Hospital, School of Medicine, Tongji University, Shanghai, China; 2 Shanghai Key Laboratory of Maternal and Fetal Medicine, Shanghai First Maternity and Infant Hospital, Shanghai, China; 3 Center for Reproductive Medicine, Shanghai First Maternity and Infant Hospital, School of Medicine, Tongji University, Shanghai, China

**Keywords:** recurrent implantation failure, insulin resistance, frozen embryo transfer, metformin, pregnancy outcomes

## Abstract

**Background:**

Recurrent implantation failure (RIF) leads to a significant waste of embryos and imposes substantial physical, emotional, and financial stress on patients. Given its complex and diverse etiology, identifying the underlying causes and developing effective interventions are crucial. Previous studies have shown that insulin resistance (IR) has negative effects on reproductive health, and metformin pre-treatment helps improve the pregnancy outcomes in IR patients. However, its role in patients with RIF remains unclear, especially in those without polycystic ovary syndrome (PCOS).

**Methods:**

A retrospective cohort study was conducted. The FET cycles of RIF patients without PCOS were stratified based on the presence or absence of IR. We used the univariate and multivariate generalized estimating equations (GEE) analysis to compare pregnancy outcomes between patients with IR and without IR, as well as between metformin-exposed and metformin-unexposed groups of RIF patients with IR.

**Results:**

In a subgroup of 941 cycles without IR and 145 cycles with IR, we found that patients with IR had a lower live birth rate (10.34% *vs* 20.94%, *P* = 0.0039) and a higher early miscarriage rate (52.77% *vs* 27.52%, *P* = 0.0034). After adjusting for potential confounders, the IR group still had a lower live birth rate (aOR = 0.5, 95% CI: 0.28-0.89, *P* = 0.019). In the subgroup of IR patients (n=330 cycles), patients in the metformin-exposed group (n=185 cycles) had a higher clinical pregnancy rate (43.24% *vs* 24.83%, *P* < 0.001), implantation rate (33.22% *vs* 17.04%, *P* < 0.001) and live birth rate (33.51% *vs* 10.34%, *P* < 0.001), as well as a lower early miscarriage rate (12.50% *vs* 52.78%, *P* < 0.01), compared to the metformin-unexposed group (n=145 cycles). These differences remained significant after adjusting for potential confounders using GEE analysis.

**Conclusions:**

Our results demonstrated that IR may be a risk factor for a low live birth rate in RIF patients without PCOS. However, the negative impact of IR on the live birth rate can be alleviated by metformin pre-treatment before FET cycles.

## Introduction

*In vitro* fertilization⁃embryo transfer (IVF⁃ET) offers infertile couples a chance to conceive their biological children. However, approximately 10%–20% of patients experience recurrent implantation failure (RIF) (1), characterized by the failure to achieve a clinical pregnancy after transferring high-quality embryos in multiple transfer cycles. The emotional and financial burden of RIF is considerable, highlighting the need for a detailed investigation into its underlying causes and potential therapeutic interventions. Nevertheless, the precise mechanisms remain inadequately understood, and effective interventions for RIF patients are limited. Currently, there are no standardized criteria for diagnosing RIF. A recent comprehensive survey defined RIF as the failure to achieve a clinical pregnancy after two or three transfers with good-quality embryos (2, 3). Evidence suggests that RIF has a multifactorial etiology, including maternal factors, embryonic factors, unhealthy lifestyle, and unknown causes (4). Among these, insulin resistance (IR) has emerged as a critical area of investigation, particularly in its impact on reproductive health (5).

IR is characterized by decreased sensitivity to exogenous or endogenous insulin, resulting in compensatory hyperinsulinemia to maintain metabolic homeostasis (6). IR and associated hyperinsulinemia may contribute to the reproductive and endocrine features of polycystic ovary syndrome (PCOS) by disrupting androgen and gonadotrophin secretion (7). This disruption may worsen reproductive and metabolic outcomes in women with PCOS undergoing ovulation induction and potentially impact pregnancy outcomes (8). IR is also a risk factor for spontaneous abortion in PCOS women undergoing IVF-ET (9). Notably, studies have shown that 23.8% of women without PCOS are also diagnosed with IR (10). However, the effects of IR on pregnancy outcomes in non-PCOS women undergoing IVF-ET remain debated (11–13). Further research is necessary to clarify its implications on reproductive outcomes in this population.

Recent research has also explored potential therapeutic interventions targeting IR to improve reproductive outcomes. Lifestyle modifications, including dietary changes and increased physical activity, enhance insulin sensitivity and restore hormonal balance in women with IR (14). Additionally, pharmacological treatments such as metformin, commonly used to improve insulin sensitivity, have been widely studied for managing PCOS (15–17). Adjunct metformin therapy could be used before and/or during FSH ovarian stimulation in women with PCOS undergoing IVF/ICSI treatment with a GnRH agonist long protocol, to reduce the risk of developing ovarian hyperstimulation syndrome and miscarriage (18). Although previous research has shown that metformin pre-treatment can improve ongoing pregnancy and implantation rates in non-PCOS women receiving IVF-ET (19), its clinical effectiveness in these patients remains controversial. A randomized double-blind controlled trial (RCT) on metformin pre-treatment for patients undergoing *in vitro* fertilization/intracytoplasmic sperm injection-embryo transfer (IVF/ICSI-ET) showed no difference in the implantation rate, miscarriage rate, or live birth rate (LBR) (20). Further investigation is needed to elucidate how IR affects reproductive outcomes in this population and to explore the potential benefits of metformin pre-treatment on fertility outcomes.

In the current study, we aim to evaluate the impact of IR status on pregnancy outcomes in non-PCOS RIF patients undergoing frozen embryo transfer (FET) cycles. Additionally, we aim to assess whether metformin pre-treatment could improve pregnancy outcomes in non-PCOS RIF patients diagnosed with IR.

## Materials and methods

### Study design and participants

This retrospective cohort study was conducted at Shanghai First Maternity and Infant Hospital. Data for this study were obtained from the hospital’s electronic database, containing all medical records of patients who underwent IVF/ICSI-ET treatments. This study was approved by the Ethics Committee of the Shanghai First Maternity and Infant Hospital (NO.KS25210), and informed consent was waived because of its retrospective design.

We screened the intact medical records of FET cycles conducted at the Center for Reproductive Medicine, and of the various examinations and treatments for RIF patients at the Department of Reproductive Immunology in our hospital from January 2019 to December 2023. Patients were enrolled in the study if they met the following criteria: (i) they were diagnosed with RIF; (ii) their records included fasting blood sugar and insulin values. Exclusion criteria were as follows: (i) preimplantation genetic testing (PGT) cycles, or cycles in which more than 50% of the blastomeres are absent; (ii) patients aged > 40 years; (iii) diagnosed with PCOS according to Rotterdam criterion (21); (iv) abnormal thyroid function or prolactin levels; (v) history of recurrent spontaneous abortion (22); (vi) patients with autoimmune diseases; and (vii) abnormal anatomy including untreated hydrosalpinx, endometrial polyps, genital tuberculosis and chronic endometritis; (viii) with missing data.

### Diagnosis

RIF was defined as a failure to achieve a clinical pregnancy despite transferring good-quality embryos under the following conditions: (1) three or more transfer cycles; or (2) at least three embryos transferred within two cycles, with at least one high-quality blastocyst or two high-quality cleavage-stage embryos transferred in each cycle. Consistent with previous studies, a good-quality blastocyst was defined as a blastocyst at stage 3 or higher, with neither inner cell mass nor trophectoderm graded C by the Gardner score system. A good-quality cleavage-stage embryo on day 3 was defined as an embryo with 7–12 blastomeres, rated as grade I-II or compacted (23). For the following analysis, we assign the group that transfers at least one high-quality embryo as Group A, while the others will be classified as Group B.

IR was diagnosed using the homeostasis model assessment for insulin resistance (HOMA-IR), calculated as [fasting insulin (µIU/mL) × fasting glucose (mmol/L)]/22.5 (24). The cutoff value for HOMA-IR was 2.71 (25).

PCOS was diagnosed based on the Rotterdam criterion (21), which require at least two of the following three conditions: chronic ovulatory dysfunction or oligomenorrhea, hyperandrogenism, and the presence of polycystic ovaries on ultrasound.

### Metformin exposure

For RIF patients with IR, those who received metformin for ≥2 months before FET were classified into the metformin-exposed group, clinicians adjusted the dosage based on body mass index (BMI), IR severity and gastrointestinal tolerance. Those who did not receive any insulin-lowering medication were classified as the metformin-unexposed group. Metformin was discontinued upon pregnancy confirmation.

### Endometrial preparation and FET

As previously described (26–29), endometrial preparation was performed in a natural cycle, ovarian induction cycle, or hormone replacement therapy based on the patient’s situation and physicians’ preference. Cleavage-stage embryo transfer was performed on the third day after ovulation or the fourth day after progesterone administration, whereas blastocyst transfer was scheduled on the fifth day after ovulation or the sixth day after progesterone administration. One or two embryos were transferred in each FET cycle.

Progesterone supplementation was continued until 10 weeks of gestation once pregnancy was confirmed. Serum beta-human chorionic gonadotropin (β-hCG) levels were measured two weeks after embryo transfer. Patients with a positive test result underwent a transvaginal ultrasonographic examination an additional two weeks later to confirm the presence of an intrauterine gestation sac with a fetal heartbeat.

### Outcome measurement

The primary outcome of this study was the LBR per FET cycle. Secondary outcomes included the biochemical pregnancy rate, clinical pregnancy rate, multiple pregnancy rate, implantation rate, early miscarriage rate, and late miscarriage rate. Serum β-hCG levels were measured 14 days after embryo transfer. For women with positive results (β-hCG ≥ 10 mIU/mL), transvaginal ultrasonography was routinely conducted 4 weeks after embryo transfer. Biochemical pregnancy was defined as a pregnancy confirmed only by the detection of β-hCG in the serum, which did not progress to a clinical pregnancy. Clinical pregnancy was defined as a pregnancy confirmed through ultrasonographic detection of one or more gestational sacs, including ectopic pregnancies. The implantation rate was calculated as the number of gestational sacs divided by the number of embryos transferred. Early miscarriage was the loss of a clinical pregnancy before the 12th week of gestation, while late miscarriage occurred after 12 weeks. Multiple pregnancy was defined as two or more gestational sacs detected by ultrasonography. A live birth was defined as the delivery of at least one live-born baby. For neonatal outcomes, low birth weight was defined as a birth weight of less than 2,500g.

### Statistical analysis

All statistical analyses were conducted using R (http://www.R-project.org) (v.4.2.3). *P* < 0.05 was considered statistically significant. Continuous variables were presented as means with standard deviation (SD), and categorical variables were presented as counts with percentages. Statistical comparison was performed using the Mann–Whitney U-test for continuous variables. Differences in categorical variables between the two groups were compared using Fisher’s exact tests or the Chi-square test as appropriate.

We used the geeglm function from the geepack package (v.1.3.9) to perform the univariate and multivariate generalized estimating equations (GEE) analyses. Both crude odds ratio (OR) and adjusted odds ratio (aOR) with a 95% confidence interval (CI) were calculated using GEE.

According to the objectives of our study, it was divided into two parts. In part one, we employed GEE analysis to examine the effect of IR on pregnancy outcomes by comparing 941 FET cycles without IR to 145 FET cycles with IR, all without metformin exposure. The analysis adjusted for confounders, including maternal age, infertility duration, BMI, number of transferred embryos, endometrial thickness on the day of embryo transfer, endometrial preparation regimen, type of infertility, and infertile factors.

In part two, we used GEE analysis to investigate the impact of metformin pre-treatment on pregnancy outcomes by comparing 145 FET cycles without metformin exposure to 185 FET cycles with metformin exposure, with both groups diagnosed with IR based on the HOMA-IR cutoff values. The analysis adjusted for confounders, including the maternal age, infertility duration, BMI, number of transferred embryos, endometrial thickness on the embryo transfer day, endometrial preparation regimen, type of infertility, infertile factors, and HOMA-IR values.

## Results

### Study groups

**Figure 1:**
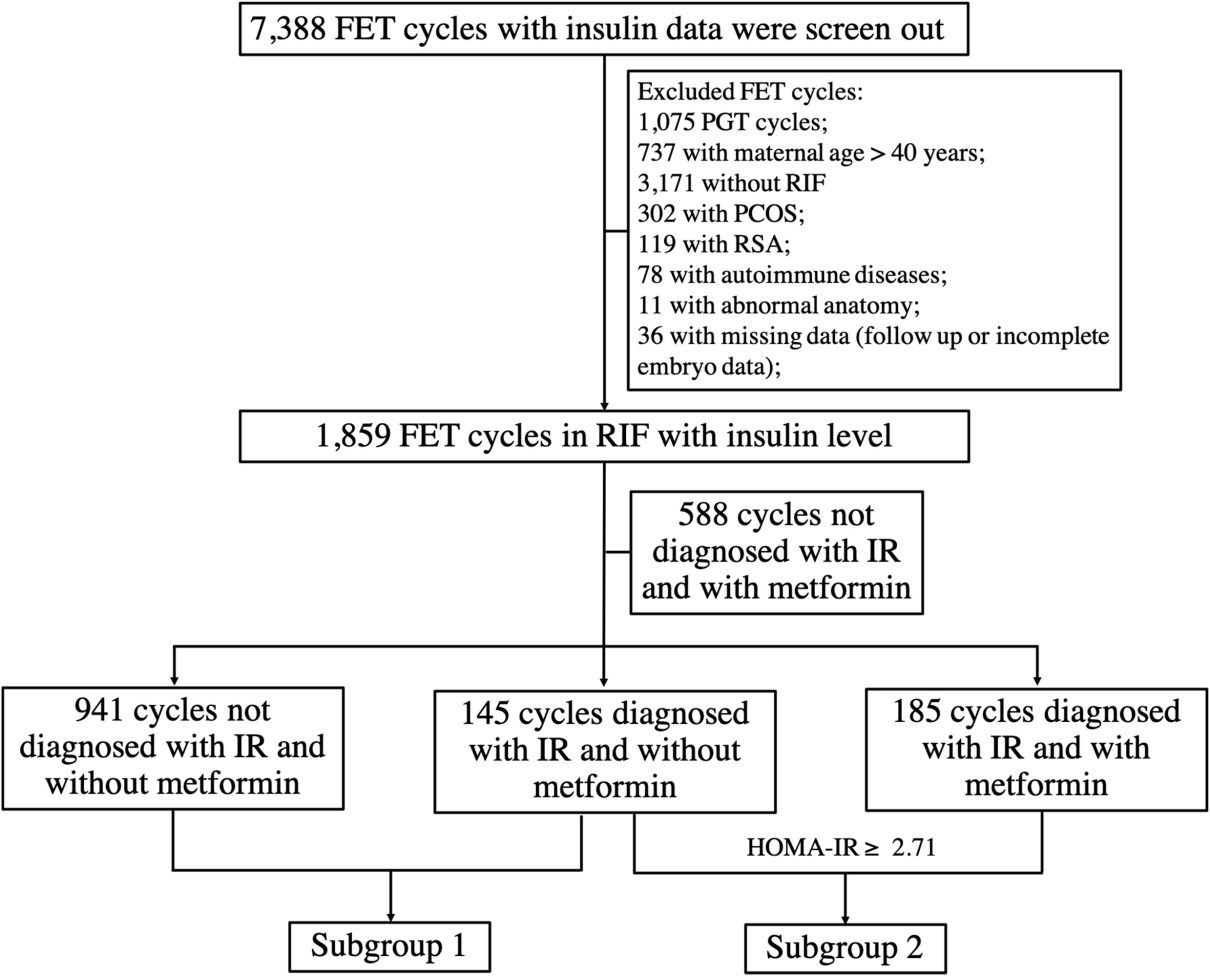
The flowchart of the study. RSA, recurrent spontaneous abortion; PGT, preimplantation genetic testing; PCOS, polycystic ovary syndrome.

The flowchart of the study is presented in **Figure 1**. After applying the inclusion and exclusion criteria, a total of 1,859 FET cycles were enrolled in the study. Of those, 1,529 cycles were diagnosed as non-IR, while 330 cycles were diagnosed as IR according to the HOMA-IR cutoff value (HOMA-IR ≥ 2.71). Among the 330 cycles, 145 cycles were unexposed to metformin, while 185 FET cycles were exposed to metformin.

### Subgroup analysis based on IR diagnosed

Initially, we investigated the role of IR in pregnancy outcomes of non-PCOS RIF patients. Among 1,086 FET cycles without metformin pre-treatment, 99 patients (145 cycles) had IR (IR group), while 605 patients (941 cycles) did not (non-IR group).

The baseline characteristics of this subgroup were summarized in **Table 1**. No significant differences were observed between the IR and non-IR groups regarding maternal age, duration of infertility, type of infertility, number of transferred embryos per FET cycle, embryo quality, or endometrial thickness on the embryo transfer day (*P* > 0.05). However, significant differences were observed in BMI, HOMA-IR values, infertility diagnosis, and the endometrial preparation regimens (*P* < 0.05) (**Table 1**).

**Table 1:** Baseline characteristics of RIF women undergoing FET in the IR and non-IR group.

Variables	IR	Non-IR	*P*
Cycles, n	145	941	NA
Patients, n	99	605	NA
Maternal age, y	33.30 ± 4.03	33.24 ± 3.51	0.81
Body mass index, kg/m^2^	24.19 ± 3.20	21.16 ± 2.62	< 0.001
Infertility duration, y	3.11 ± 1.98	3.25 ± 2.48	0.66
No. of transferred embryos per FET cycle, n	1.54 ± 0.50	1.56 ± 0.50	0.55
Endometrial thickness, mm	9.96 ± 2.16	9.76 ± 2.08	0.37
HOMA-IR	4.10 ± 1.60	1.41 ± 0.56	< 0.001
Type of infertility, n (%)			0.38
Primary infertility	83 (57.24)	498 (52.92)	
Secondary infertility	62 (42.76)	443 (47.08)	
Infertility diagnosis, n (%)			0.028
Female factor	85 (58.62)	589 (62.59)	
Male factor	20 (13.79)	176 (18.70)	
Combination of factors	28 (19.31)	104 (11.05)	
Unknown factor	12 (8.28)	72 (7.65)	
Endometrial preparation regimen, n (%)			0.023
Natural cycle	21 (14.48)	190 (20.19)	
Ovarian induction cycle	19 (13.10)	181 (19.23)	
Hormone replacement therapy	105 (77.21)	570 (60.57)	
Number of embryos transferred, n (%)			0.6
SET	67 (46.21)	410 (43.57)	
DET	78 (53.79)	531 (56.43)	
Type of embryo transfer, n (%)			0.30
Cleavage embryo transfer	80 (55.17)	577 (61.32)	
Blastocyst transfer	61 (42.07)	347 (36.88)	
sequential embryo transfer	4 (2.76)	17 (18.07)	
Embryo quality, n (%)			0.92
A	104 (71.72)	670 (71.20)	
B	41 (28.28)	271 (28.80)	

n, number; y, years; FET, frozen embryo transfer; RIF, recurrent implantation failure; IR, insulin resistance; HOMA-IR, homeostasis model assessment of insulin resistance; NA, not available; SET, single embryo transfer; DET, double embryo transfer; Sequential embryo transfer refers to transfer one frozen-thawed cleavage embryo followed one frozen-thawed blastocyst.

Pregnancy and neonatal outcomes were presented in **Table 2**; **Supplementary Figure 1**. No significant differences were observed in biochemical pregnancy rate, clinical pregnancy rate, multiple pregnancy rate, implantation rate, ectopic pregnancy rate, or late miscarriage rate (*P* > 0.05). However, the IR group had a lower LBR (10.34% *vs* 20.94%, *P* = 0.0039) and a higher early miscarriage rate (52.77% *vs* 27.52%, *P* = 0.0034), compared to non-IR group. Neonatal outcomes revealed that IR and non-IR patients were similar in terms of gestational weeks, preterm birth rate, proportion of singletons, percentage of low birthweight newborns, and incidence of congenital malformations (*P* > 0.05).

**Table 2:** Pregnancy and neonatal outcomes of RIF women undergoing FET in the IR and non-IR group.

Variables	IR	Non-IR	*P*
Cycles, n	145	941	NA
Pregnancy outcomes of FET, n (%)			
Biochemical pregnancy rate	15 (10.35)	88 (9.35)	0.82
Clinical pregnancy rate	36 (24.83)	298 (31.67)	0.12
Multiple pregnancy rate	3 (8.33)	44 (14.77)	0.45
Implantation rate	38/223 (17.04)	336/1,472 (22.83)	0.064
Ectopic pregnancy rate	1 (2.77)	9 (3.02)	1
Early miscarriage rate	19 (52.77)	82 (27.52)	0.0034
Late miscarriage rate	1 (2.77)	10 (3.36)	1
Live birth rate	15/145 (10.34)	197/941 (20.94)	0.0039
Gestational weeks, w	38.10 ± 2.64	38.07 ± 2.44	0.66
Live birth cycles, n (%)			0.71
Preterm delivery	2 (13.33)	39 (19.80)	
Term delivery	13 (86.67)	158 (80.20)	
Live born infants, n (%)			0.71
Single newborn, n (%)	12 (80.00)	166 (84.26)	
Twin newborns, n (%)	3 (20.00)	31 (15.74)	
Single birth weight, g	3461.67 ± 321.62	3247.49 ± 613.49	0.27
Newborn with birth weight < 2500 g, n (%)	5 (27.78)	44 (19.30)	0.56
Congenital malformations, n (%)	1 (5.56)	8 (3.51)	0.50

n, number; y, years; w, week; FET, frozen embryo transfer; mm, millimeter; g, gram; NA, not available.

To further explore the association between IR and LBR and early miscarriage rate, we conducted univariate and multivariate GEE analyses (**Table 3**). Both univariate and multivariate GEE analyses revealed no significant differences in early miscarriage rate (*P* > 0.05). However, the univariate GEE analysis showed that IR group had a lower LBR (OR 0.44, 95% CI: 0.25-0.76, *P* = 0.0032) (**Table 3**). After adjusting for potential confounders, including the maternal age, infertility duration, BMI, number of transferred embryos, endometrial thickness on the embryo transfer day, endometrial preparation regimen, type of infertility, and infertile factors in the multivariable GEE model, IR group still remained a lower LBR (aOR 0.50, 95% CI 0.28-0.89, *P* = 0.019) (**Table 3**; **Supplementary Table 1**).

**Table 3:** Univariate and multivariate generalized estimating equations analyses results of the association between IR status and clinical outcomes.

Variables	Groups	OR (95% CI)	*P*	aOR (95% CI)	*P*
Early miscarriage rate	Non-IR	Reference			
	IR	1.58 (0.89-2.79)	0.12	1.47 (0.80-2.68)	0.22
LBR	Non-IR	Reference			
	IR	0.44 (0.25-0.76)	0.0032	0.50 (0.28-0.89)	0.019

Analyses were adjusted for maternal age, BMI, endometrial preparation regimen, type of infertility, Infertility diagnosis, Infertility duration, number of transferred embryos per FET cycle, and endometrial thickness; OR, odds ratio; aOR, adjusted odds ratio; CI, confidence interval.

### Subgroup analysis based on metformin exposure

Next, we tried to explore whether metformin pre-treatment would improve the pregnancy outcomes in non-PCOS patients with IR. A total of 330 transfer cycles were involved in the analysis. Among these, 130 patients who completed 185 transfer cycles had been exposed to metformin (Metformin group) prior to FET, while 99 patients underwent 145 transfer cycles unexposed to metformin (Non-metformin group) prior to FET.

The baseline characteristics of this subgroup were presented in **Table 4**. Except for the endometrial preparation regimen, which showed a statistical difference between the two groups, (*P* = 0.0078), no significant differences were observed in maternal age, BMI, duration of infertility, number of transferred embryos per FET cycle, embryo quality, endometrial thickness, HOMA-IR, type of infertility, infertility diagnosis, number of embryos transferred, or type of embryo transfer between the two groups (*P* > 0.05). Pregnancy and neonatal outcomes indicated that Metformin group was associated with a higher clinical pregnancy rate (43.24% *vs* 24.83%, *P <* 0.001), implantation rate (32.22% *vs* 17.04%, *P <* 0.001), and LBR (33.51% *vs* 10.34%, *P <* 0.001), as well as a lower early miscarriage rate (12.50% *vs* 52.78%, *P < 0.001*) (**Table 5**; **Supplementary Figure 2**). However, no statistical differences were found in other indicators, such as biochemical pregnancy rate, ectopic pregnancy rate, and late miscarriage rate (*P* > 0.05). Additionally, there were no statistical differences in neonatal outcomes, including preterm birth rate, multiple pregnancy rate, singleton birth weight, and congenital malformation rate (*P* > 0.05).

**Table 4:** Baseline characteristics of RIF women undergoing FET in the metformin and non-metformin group.

Variables	Metformin	Non-metformin	*P*
Cycles, n	185	145	NA
Patients, n	130	99	NA
Maternal age, y	33.36 ± 3.43	33.30 ± 4.03	0.85
Body mass index, kg/m^2^	24.23 ± 3.22	24.19 ± 3.19	0.96
Infertility duration, y	3.30 ± 2.52	3.11 ± 1.98	0.93
No. of transferred embryos per FET cycle, n	1.56 ± 0.50	1.54 ± 0.50	0.66
Endometrial thickness, mm	9.73 ± 2.33	9.96 ± 2.16	0.21
HOMA-IR	4.22 ± 2.03	4.10 ± 1.60	0.39
Type of infertility, n (%)			0.51
Primary infertility	98 (52.97)	83 (57.24)	
Secondary infertility	87 (47.03)	62 (42.76)	
Infertility diagnosis, n (%)			0.097
Female factor	128 (69.19)	85 (58.62)	
Male factor	22 (11.89)	20 (13.79)	
Combination of factors	19 (10.27)	28 (19.31)	
Unknown factor	16 (8.65)	12 (8.28)	
Endometrial preparation regimen, n (%)			0.0078
Natural cycle	20 (10.81)	21 (14.48)	
Ovarian induction cycle	50 (27.03)	19 (13.10)	
Hormone replacement therapy	115 (62.16)	105 (72.41)	
Number of embryos transferred, n (%)			0.7
SET	81 (43.78)	67 (46.21)	
DET	104 (56.21)	78 (53.79)	
Type of embryo transfer, n (%)			0.3
Cleavage embryo transfer	114 (61.62)	80 (55.17)	
Blastocyst transfer	69 (37.30)	61 (42.07)	
Sequential embryo transfer	2 (1.08)	4 (2.76)	
Embryo quality, n (%)			0.90
A	135 (72.97)	104 (71.72)	
B	50 (27.03)	41 (28.28)	

n, number; y, years; FET, frozen embryo transfer; RIF, recurrent implantation failure; IR, insulin resistance; HOMA-IR, homeostasis model assessment of insulin resistance; NA, not available; SET, single embryo transfer; DET, double embryo transfer; Sequential embryo transfer refers to transfer one frozen-thawed cleavage embryo followed one frozen-thawed blastocyst.

**Table 5:** Pregnancy and neonatal outcomes of RIF women undergoing FET in the metformin and non-metformin group.

Variables	Metformin	Non-metformin	*P*
Cycles, n	185	145	NA
Pregnancy outcomes of FET, n (%)			
Biochemical pregnancy rate	16 (8.65)	15 (10.34)	0.74
Clinical pregnancy rate	80 (43.24)	36 (24.83)	< 0.001
Multiple pregnancy rate	17 (21.25)	3 (8.33)	0.10
Implantation rate	96/289 (33.22)	38/223 (17.04)	< 0.001
Ectopic pregnancy rate	3 (3.75)	1 (2.78)	1
Early miscarriage rate	10 (12.50)	19 (52.78)	< 0.001
Late miscarriage rate	5 (6.25)	1 (2.78)	0.66
Live birth rate	62/185 (33.51)	15/145 (10.34)	< 0.001
Gestational weeks, w	38.31 ± 1.96	38.10 ± 2.64	0.76
Live birth cycles, n (%)			
Preterm delivery	10 (16.13)	2 (13.33)	1
Term delivery	52 (83.87)	13 (86.67)	
Live born infants, n (%)			0.71
Single newborn	52 (83.87)	12 (80.00)	
Twin newborns	10 (16.13)	3 (20.00)	
Single birth weight, g	3440.48 ± 511.55	3461.67 ± 321.62	0.80
Newborn with birth weight < 2500 g, n (%)	10 (13.89)	5 (27.78)	0.29
Congenital malformations, n (%)	4 (5.63)	1 (6.25)	1

n, number; y, years; w, week; FET, frozen embryo transfer; mm, millimeter; g, gram; NA, not available.

Then, we assessed the association between the metformin pre-treatment and the clinical pregnancy rate, implantation rate, LBR, and early miscarriage rate using univariate and multivariable GEE models. In the univariate GEE model, metformin pre-treatment was significantly associated with higher clinical pregnancy rate (OR 2.31, 95% CI: 1.43-3.72, *P* < 0.001), implantation rate (OR 2.31, 95% CI 1.43-3.72, *P* < 0.001), and LBR (OR 4.37, 95% CI: 2.38-8.01, *P* < 0.001), as well as lower early miscarriage rate (OR 0.38, 95% CI: 0.16-0.88, *P* = 0.024) (**Table 6**). After adjusting for potential confounders, such as maternal age, infertility duration, BMI, number of transferred embryos, endometrial thickness on the embryo transfer day, endometrial preparation regimen, type of infertility, infertile factors, and HOMA-IR values, metformin pre-treatment remained associated with reduced risk of early miscarriage rate (aOR 0.35, 95% CI: 0.15-0.84, *P* = 0.016) and increased likelihood of clinical pregnancy rate (aOR 2.40, 95% CI: 1.47-3.92, *P* < 0.001), implantation rate (aOR 2.40, 95% CI: 1.47-3.92, *P* < 0.001), and LBR (aOR 4.90, 95% CI 2.58-9.29, *P* < 0.001) (**Table 6** and **Supplementary Table 2**).

**Table 6:** Univariate and multivariate generalized estimating equations analyses results of the association between metformin status and clinical outcomes.

Variables	Groups	OR (95% CI)	*P*	aOR (95% CI)	*P*
Clinical pregnancy rate	Non-metformin	Reference			
	Metformin	2.31 (1.43-3.72)	< 0.001	2.40 (1.47-3.92)	< 0.001
Implantation rate	Non-metformin	Reference			
	Metformin	2.31 (1.43-3.72)	< 0.001	2.40 (1.47-3.92)	< 0.001
Early miscarriage rate	Non-metformin	Reference			
	Metformin	0.38 (0.16-0.88)	0.024	0.35 (0.15-0.84)	0.016
LBR	Non-metformin	Reference			
	Metformin	4.37 (2.38-8.01)	< 0.001	4.90 (2.58-9.29)	< 0.001

Analyses were adjusted for maternal age, endometrial preparation regimen, type of infertility, BMI, Infertility diagnosis, Infertility duration, HOMA-IR, number of transferred embryos per FET cycle, and endometrial thickness; OR, odds ratio; aOR, adjusted odds ratio; CI, confidence interval.

## Discussion

RIF is a complex condition with multiple causes (e.g., immunology, thrombophilias, endocrine disorders, metabolic dysregulation, microbiome alterations, anatomical defects, male factors, and genetics), posing significant challenges for patients and clinicians (30). This study investigated the association between IR and pregnancy outcomes in non-PCOS RIF patients and the effect of metformin pre-treatment in IR patients. We found that IR is a risk factor for reduced LBR in non-PCOS patients. However, metformin pre-treatment for ≥2 months before FET mitigated this effect, leading to improvements in clinical pregnancy, implantation rate, and LBR, while also reducing early miscarriage rate. Our findings highlight the detrimental effects of IR and support metformin pre-treatment for improving outcomes.

IR is a metabolic condition characterized by the diminished ability of cells to respond effectively to insulin. This phenomenon is increasingly prevalent among women of reproductive age (31). The rising incidence of IR among women has significant implications for reproductive health, as it is closely linked to various endocrine disorders, including PCOS, infertility, and other reproductive dysfunctions (32). IR is thought to contribute to adverse outcomes in ART cycles, including lower pregnancy rate, reduced implantation rate, and higher miscarriage rate (25, 33, 34). The mechanisms underlying the impact of IR on adverse pregnancy outcomes are multifaceted. In addition to the impact on reproductive hormones and ovulation, studies have shown that women with IR often experience endometrial abnormalities, which can increase the risk of adverse pregnancy outcomes (35). Furthermore, IR is associated with chronic low-grade inflammation, which can adversely affect ovarian function, endometrial receptivity, and live birth (36).

Some studies have shown that as HOMA-IR values increased, the LBR markedly decreased across different PCOS groups (37) and that IR is significantly associated with LBR in fresh ET cycles in women with PCOS (38). However, the correlation between IR and the LBR in assisted reproduction is still controversial. A study by Luo et al. found that IR had no significant effect on pregnancy rate and LBR in the first fresh embryo transfer cycles (39). Among non-PCOS subjects, previous studies have reported that hyperinsulinemia and IR have no impact on the reproductive outcomes in women undergoing assisted reproduction (13). However, a recent study has confirmed that in the context of ART, infertile non-PCOS women with IR showed a higher clinical miscarriage, leading to fewer live births compared with the insulin-sensitive infertile patients (40). In our study, when comparing the pregnancy outcomes between IR and non-IR groups, we also found that patients diagnosed with IR had a lower LBR, even after adjusting for the potential confounders. This suggests that IR may be a risk factor for low LBR. Thus, our findings highlight the importance of managing IR in women undergoing fertility treatment to improve reproductive outcomes.

In clinical practice, the treatment of RIF is also very tricky. To improve treatment efficacy for individuals facing RIF with IR, we pay attention to whether pre-treating IR with metformin improves pregnancy outcomes in RIF patients. However, the effect of metformin pre-treatment in ART remains debated. A 2020 meta-analysis of women with PCOS found no conclusive evidence that metformin improves LBR (41). Additionally, an RCT analysis also showed no difference in implantation rate, miscarriage rate, or LBR with or without metformin pre-treatment (20). Moreover, studies have shown that metformin can improve ongoing pregnancy, implantation rates, and LBR, as well as lower clinical miscarriage (19, 40). We found that IR patients with metformin pre-treatment showed higher clinical pregnancy rate, implantation rate, and LBR, along with lower early miscarriage rate. The significant difference still remained in the GEE model. These results emphasize the need for further studies to confirm the effects of metformin pre-treatment on pregnancy outcomes in ART.

Metformin can suppress appetite to reduce weight and plasma insulin levels. It can decrease hyperandrogenism and improve menstrual cycles and ovulation in women with PCOS (42, 43). Metformin has been shown to enhance SLC2A4 function in the endometrium, which is the crucial role of the insulin-dependent glucose transporter SLC2A4 in endometrial glucose uptake, and a low expression of SLC2A4 can impair endometrial metabolism (9). Additionally, other studies suggested that although embryonic development may not be directly affected by IR, endometrial receptivity was often compromised, which may reduce the implantation rate and LBR (44). Metformin appears to improve endometrial receptivity, thereby enhancing both implantation rate and LBR (45). A recent study using spatial transcriptome sequencing and single-cell transcriptome sequencing techniques indicated that 16 weeks of metformin treatment can improve the health of the endometrium in PCOS patients by targeting integrin signaling and dysregulated pathways (46). It was also reported that metformin could improve dyslipidemia in a non-PCOS population of patients (47); therefore, it is evident that metformin has potential benefits for non-PCOS patients. Since there is little research on non-PCOS at present, we hypothesize that similar to the PCOS group, metformin may improve the pregnancy outcomes by improving endometrial function in the non-PCOS group, but further studies are still needed to verify it.

Effective endometrial preparation before FET is crucial for synchronizing the embryonic and endometrial windows of receptivity, which is key to achieving successful implantation. This preparation can be achieved through a natural cycle, an ovarian induction cycle, or a hormone replacement therapy. In the current study we found statistical differences between IR and non-IR group, as well as between metformin and non-metformin group in terms of endometrial preparation regimens (**Tables 1** and **4**). To assess the potential impact of different regimens on the study outcomes, we performed a GEE analysis. Both univariate and multivariate GEE analyses confirmed that the endometrial preparation regimens had no effect on the conclusions of the current study. This aligns with previously reported findings, which suggest that different endometrial preparation regimens do not impact clinical outcomes (48, 49). However, the possibility of residual confounders cannot be ruled out. For example, due to the absence of corpus luteum in HRT, is likely contributing to the increased risk of obstetric complications and hypertensive disorders of pregnancy (50, 51). Since progesterone is primarily secreted by the corpus luteum, it remains unclear whether the lack of an endogenous progesterone could influence outcomes of assisted reproductive treatments. This highlights the need for further studies involving larger cohorts to explore more uniform endometrial preparation regimens or to stratify by preparation regimens to minimize variability and provide clearer insights into the current conclusions.

Our study provides novel insights into how IR influences pregnancy outcomes and how metformin treatment for IR enhances reproductive outcomes in women with RIF who do not have PCOS before FET. However, some limitations still exist. First, the small sample size means our findings should be interpreted with caution. Future research should evaluate the effects of metformin treatment in larger, independent cohorts of RIF patients without PCOS. Additionally, incomplete data on fasting plasma glucose and insulin levels after metformin treatment further limit the strength of our findings. Future studies should include prospective trials with larger cohorts to assess the impact of metformin on FET outcomes in patients with RIF and IR. Furthermore, due to the retrospective nature of our study, we cannot fully eliminate the potential impact of sample selection bias, embryonic factors, and the information regarding the embryos transferred in previous IVF cycles of RIF patients on the conclusions, which may limit the generalizability of the conclusions. Therefore, future prospective cohort studies are needed to validate the findings of this study further. Lastly, women with IR who are exposed to metformin may be more attentive to their health status and, as a result, may pursue additional healthcare interventions to improve the success of FET. This combined effect suggests that the improvement in clinical outcomes for RIF patients with IR may not be solely attributed to the effect of metformin. This represents another limitation of the study that should be addressed in future research.

## Conclusions

In conclusion, our study highlights the detrimental effect of IR on the pregnancy outcomes in non-PCOS patients with RIF and the beneficial effects of metformin treatment before FET on pregnancy outcomes in patients with RIF without PCOS. Our findings support the importance of addressing IR during IVF/ICSI treatments. Identifying women at high risk of IR is crucial for optimizing preventative and therapeutic strategies in patients with RIF. However, more large-scale prospective studies are warranted to ascertain the benefits of metformin on pregnancy outcomes and determine the optimal dose and duration of metformin treatment based on the severity of IR.

## Data Availability

The original contributions presented in the study are included in the article/**Supplementary Material**. Further inquiries can be directed to the corresponding author.
