# Supplementary material for: Metformin improves pregnancy outcomes in non-PCOS women with insulin resistance and recurrent implantation failure before frozen embryo transfer
Source: Front Endocrinol (Lausanne). 2025 Dec 10;16:1671899. doi: 10.3389/fendo.2025.1671899 (PMC12727630; doi:10.3389/fendo.2025.1671899)
Supplement: Supplementary file 1 [file Table1.docx]

**Supplemental Materials**

**Supplementary Figure 1. Clinical outcomes between the IR and non-IR groups during the FET cycles.** ** *P* < 0.01; * *P* < 0.05.

**Supplementary Figure 2. Clinical outcomes between the Metformin and Non-metformin groups during the FET cycles.** *** *P* < 0.001; * *P* < 0.05

**Supplementary Table 1 Statistical results of adjusted variables in the multivariate GEE analysis examining the association between IR status and clinical outcomes**

| Variables | *P* value | |
| --- | --- | --- |
|  | Early miscarriage (OR 95% CI) | LBR (OR 95% CI) |
| Maternal age | 0.319 | < 0.001 (0.93 0.89-0.97) |
| BMI | 0.987 | 0.34 |
| Endometrial preparation regimen | 0.017 (1.49 1.07-2.05) | < 0.001 (0.71 0.59-0.86) |
| Type of infertility | 0.121 | 0.14 |
| Infertility diagnosis | 0.345 | 0.47 |
| Infertility duration | 0.627 | 0.37 |
| NO. of transferred embryos | 0.232 | 0.097 |
| Endometrial thickness | 0.362 | 0.14 |

Note: BMI: Body mass Index; LBR: live birth rate. OR: odds ratio; CI: confidence interval.

**Supplementary Table 2 Statistical results of adjusted variables in the multivariate GEE analysis examining the association between metformin status and clinical outcomes**

| Variables | *P* value | | | |
| --- | --- | --- | --- | --- |
|  | Early miscarriage (OR 95% CI) | LBR (OR 95% CI) | Clinical pregnancy (OR 95% CI) | Implantation (OR 95% CI) |
| Maternal age | 0.69 | 0.058 | 0.082 | 0.82 |
| BMI | 0.89 | 0.99 | 0.76 | 0.76 |
| Endometrial preparation regimen | 0.69 | 0.40 | 0.19 | 0.19 |
| Type of infertility | 0.01 (0.28 0.10-0.74) | 0.24 | 0.87 | 0.87 |
| Infertility diagnosis | 0.32 | 0.015 (1.34 1.06-1.69) | 0.23 | 0.23 |
| Infertility duration | 0.83 | 0.19 | 0.17 | 0.17 |
| NO. of transferred embryos | 0.14 | 0.04 (1.84 1.03-3.29) | 0.0031 (2.07 1.28-3.36) | 0.0031 (2.07 1.28-3.36) |
| Endometrial thickness | 0.40 | 0.12 | 0.91 | 0.91 |
| HOMA-IR | 0.49 | 0.79 | 0.25 | 0.25 |

Note: BMI: Body mass Index; LBR: live birth rate; HOMA-IR: homeostasis model assessment for insulin resistance. OR: odds ratio; CI: confidence interval.
